# Metacognition in dogs: Do dogs know they could be wrong?

**DOI:** 10.3758/s13420-018-0367-5

**Published:** 2018-11-12

**Authors:** Julia Belger, Juliane Bräuer

**Affiliations:** 1 Max Planck Institute for the Science of Human History, Department of Linguistic and Cultural Evolution, Dogstudies, Kahlaische Strasse 10, 07745 Jena, Germany; 2 Department for General Psychology and Cognitive Neuroscience, Friedrich Schiller University Jena, Jena, Germany

**Keywords:** Metacognition, Domestic dog, Seeking information, Comparative psychology

## Abstract

In the current study, we investigated the question of whether dogs were sensitive to the information that they themselves had or had not acquired. For this purpose, we conducted three consecutive experiments in which dogs had to find a reward that was hidden behind one of two V-shaped fences with a gap at the point of the V. This setup allowed us to distinguish between selecting one of the fences by walking around it and seeking additional information by checking through the gap in the fence. We varied whether dogs had visual access to the baiting procedure or not. In addition, we manipulated the type and quality of reward as well as the time delay between baiting and choosing to analyze if the dogs’ searching behavior was affected. Our results were partly consistent with the findings of Call (Animal Cognition, 13 (5)*,* 689–700, 2010) with great apes, on whose findings we based our experiments. We found that dogs checked more often through the corner of the V-shaped fence when they had not seen where the reward was hidden. Interestingly, dogs rewarded with toys selected the correct fence more often than dogs rewarded with food. Even though dogs’ performance was not affected by the food quality condition, dogs were significantly faster in fetching a high-quality food reward as opposed to a low-quality food reward. When testing whether forgetting and checking would increase as a function of delay, we found that although dogs slightly decreased in their success in finding the food when time delays were longer, they were not more likely to check before choosing. We show that – similar to apes – dogs seek additional information in uncertain situations, but their behavior in uncertain situations is less flexible compared to great apes.

**Electronic supplementary material:**

The online version of this article (10.3758/s13420-018-0367-5) contains supplementary material, which is available to authorized users.

## General introduction

While in recent years much attention has been given to what animals understand about each other, only little is known about what animals understand about their own mental processes. Moreover, the evolutionary origins of metacognition – the ability to access, monitor, and control one’s own perceptual and cognitive processes and, thus, know about one’s own cognitive potentials as well as limitations (Flavell, 1979; Hertzog & Hultsch, 2000; Smith, Shields, & Washburn, 2003; Zohar, 1999) – are still widely debated (Rosati & Santos, 2016). The question therefore arises whether humanlike forms of metacognition exist in other species (Carruthers, 2008; Crystal & Foote, 2011; Hampton, 2009; Kornell, 2009; Smith, Beran, Couchman, & Coutinho, 2008). Thus, the general issue we raise here is whether animals have access to what they have seen and what they know, and whether they seek additional information in situations of uncertainty.

However, the question is not only whether animals share humans’ capacity for metacognition (Foote & Crystal, 2007; Smith, Shields, & Washburn, 2003; Smith, 2009), but also what the best methods are for studying non-linguistic behavior for evidence of metacognition in animals. Comparative psychologists have conducted cognitive tests on non-human animals to determine whether they possess knowledge of their own cognitive states by using memory and food concealment as well as perceptual and information-seeking paradigms (Kornell, 2014). It seems that some animals make certain judgments in similar ways to humans, although not by directly accessing their memories but rather by drawing inferences based on cues like ease of processing and reaction time (Kornell, 2014).

Griffin (2004) has emphasized that all animals regularly face uncertain situations, not only when they have to read social signals but also when they have to make a determination about the presence of a predator or available food. It is essential for survival to evaluate ambiguous information. Therefore, it is clearly advantageous to differentiate between certain and uncertain situations, paying the cost of seeking extra information only when it is really necessary (Griffin, 2004). In a number of different experiments, it has been shown that humans, dolphins, monkeys, and rats refuse to complete trials that are difficult, such as at a threshold in an auditory discrimination task. In other words, when the task is difficult, the risk of failing at a task, and therefore not receiving a reward, is so high that it might not be worth the cost of trying. In some cases, a wrong choice could, additionally, result in a time-out (rhesus macaques: Hampton, 2001; Smith et al., 2006; rats: Foote & Crystal, 2007; orangutans: Suda-King, 2008; also, see Smith et al., 2003 and Smith, 2009 for reviews). Additionally, it has been argued that subjects perform better in tests when they have the option to decline trials as compared to when they are forced to make a decision (Foote & Crystal, 2007; Hampton, 2001). These results can be interpreted as evidence for the fact that these species know what they remember (but see Browne, 2004 and Carruthers, 2008 for different interpretations).

Several researchers have criticized such methods by arguing that the results of these tests could be interpreted in an associative-behaviorist way (Smith, Zakrzewski, & Church, 2016). More precisely, in a more difficult trial an uncertainty state is created when a perceptual threshold is exceeded. By using the uncertainty response, the animal will know about knowing or not know whether it will successfully pass trials that are at the perceptual threshold (Smith, Beran, Couchman, Coutinho, & Boomer, 2009). However, studies using uncertainty responses have been criticized because the animals’ behavior might solely be based on learned responses to a specific stimulus (Carruthers, 2008; Crystal & Foote, 2009).

Call and Carpenter (2001) introduced a novel and different approach to the question of metacognition: the information-seeking paradigm that does not require extensive training or prior knowledge. The key features of this more naturalistic approach are that animals can seek additional information when needed, which enables them to respond accordingly as soon as they have gathered the relevant information (Call & Carpenter, 2001). The experimental set-up in Call and Carpenter’s study consisted of two parallel tubes that chimpanzees, orangutans, and 2.5-year-old children observed. The tubes were placed on a platform with their openings oriented towards the subjects. Then the experimenter placed a piece of food inside one of the tubes while ensuring that the subject was aware of the baiting procedure. In order to receive the reward, they had to touch the baited tube containing the bait on the first attempt. They introduced two conditions: in one condition, the subjects witnessed the baiting process (Seen condition), while in the other condition, baiting took place behind an opaque occlude that blocked the subjects’ visual access to the bait (Unseen condition). All subject groups spontaneously bent down more often to look inside the tubes before making a decision during the Unseen condition. The authors concluded that subjects had access to their own mental states (Call & Carpenter, 2001).

The information-seeking paradigm has been subject to criticism on the grounds that animals could just engage in a routine by looking for information instead of applying metacognitive abilities (Call, 2010). Call (2012) pointed out two sorts of alternative explanations of a non-metacognitive nature. One alternative non-metacognitive approach is the broad-beam explanation, which states that a non-metacognitive construct actually accounts for the observed results in studies on animal metacognition and not on monitoring processes of knowledge states. The second approach is the narrow-beam hypothesis (see Call, 2012), which claims that subjects who lack information about a reward’s location engage in search behavior until they find it. Many animals are presumably engaged in this so-called “search, locate, retrieve routine,” which might be an alternative explanation for the results in the hidden and visible trials. To address this issue, Call (2010) introduced five conditions to test the flexibility of the information-seeking behavior in great apes. He referred to the so-called “Passport Effect,” i.e., that in humans as well as in other animals, whether an individual will search for extra information depends on various factors, such as the value of the “reward” (i.e., a passport is more valuable than a tram ticket) and the time delay between hiding and searching (i.e., re-checking for the passport when it was packed yesterday, but not 5 min ago).

In his study, Call (2010) introduced five conditions, manipulating (1) whether subjects had visual access to the baiting, (2) costs associated with seeking information, (3) food quality, (4) additional information offered regarding the food’s location, and (5) the time delay between baiting and selecting one of the hiding places. Call concluded that his ape subjects knew that they could be wrong and that “the looking response appears to be a function of at least three factors: the cost of looking inside the tube, the value of the reward and the state of the information” (p. 699).

The domestic dog (*Canis familiaris*) represents an interesting model to study animal cognition as during the long domestication process dogs have evolved special skills to function effectively in the human environment, such as reading human social and communicative skills (Marshall-Pescini & Kaminski, 2014), in which they even outperform great apes (i.e., Bräuer et al., 2006; Hare et al., 2002). However, the literature has no consensus on metacognition in dogs, i.e., whether they have knowledge of their own cognitive states (Bräuer, Call, & Tomasello, 2004).

McMahon, Macpherson, and Roberts (2010) applied an information-seeking paradigm, where subjects needed to fetch a reward without immediately available information. To fetch the hidden reward, dogs had to seek additional information. More precisely, the experimental set-up was comprised of four boxes, all of which were completely black, except for one box, which had a white side. In an extensive training, dogs learned that the reward was always hidden under the box with the white side. In the experimental manipulation, the boxes were rotated (45**°**, 90**°**, and 135**°**) and, thus, the one white side gradually rotated out of the dogs’ view. Their findings show that the dogs’ accuracy progressively declined. The authors concluded that if dogs could use additional information, as stated in the information-seeking paradigm, they should have walked around the boxes in order to choose the correct one. In a follow-up experiment, the authors again applied an information-seeking paradigm, but this time in a human-oriented context, to examine whether dogs would seek further information. The reward could be hidden underneath one of three boxes. Before being able to select one of the boxes, dogs had to choose one of two human experimenters, where one was the informant (i.e., person who would point to a location) and the other was the non-informant (i.e., person who would not provide any information by turning his or her back to the dog). Dogs chose the informant significantly more often than the non-informant, which suggests that dogs seek additional information in an information-seeking task when the information source is a human (McMahon, Macpherson, & Roberts, 2010).

Similarly, Bräuer, Call, and Tomasello (2004) investigated whether dogs are sensitive to the information they themselves have acquired. In an object-choice task, dogs were presented with two identical wooden boxes, of which only one contained a baited reward. On one side of each box was a transparent window of glass with holes through which dogs could seek extra information about whether the food was placed in that box, such as by looking or smelling through the window. On the other side of that box there was a lever, and dogs were trained to select one of the boxes by pressing this lever with their paw. In the Seen condition, the location of the reward was shown to the dogs and therefore the dogs had information about the location of the food. In the Unseen condition they were prevented from seeing the baiting procedure by two occluding barriers. Before selecting, the dogs had the opportunity to seek extra information regarding the location of the hidden reward, which would be especially useful in the Unseen condition. The results showed that the dogs selected the correct box in the Seen condition, but performed only at chance level when they were prevented from seeing the reward’s location. Most importantly, dogs rarely showed checking behavior before selecting one of the boxes and they did not check more often, as assumed, in the Unseen condition compared to the Seen condition. The authors concluded that their findings might indicate that dogs do not have access to their own perceptual and knowledge states (Bräuer, Call, & Tomasello, 2004).

However, both of these studies about metacognitive abilities in dogs had some constraints. First, training was involved (i.e., pressing the lever in Bräuer et al., 2004, and learning that the food is in the box with the white side in McMahon et al., 2010), and second, dogs were rewarded with food. It is possible that dogs would show a more flexible searching behavior when they searched for their favorite toy – a precise object they “personally” know and that they fetch and that does not “disappear” as they consume it. (Note that in dog studies about object permanence and memory, toys are often used as a reward; see Collier-Baker et al., 2004; Fiset et al., 2003; Miller et al., 2009; Müller et al., 2014.)

More importantly, training could have led to an automatic response in the dogs. Thus, dogs chose a box because they had learned to do so and could not inhibit this response despite their lack of information about the contents of the boxes (Bräuer et al., 2004). Therefore, in the current study we investigated metacognition in dogs using a new set-up in which dogs did not have to learn new behaviors in order to check or to make their decision. As it is clearly adaptive to differentiate between certain and uncertain situations (see above, Griffin, 2004), and as dogs show special social cognitive skills (Marshall-Pescini & Kaminski, 2014), we hypothesized that dogs would show flexible metacognitive skills – comparable to those of apes and human children – when tested in an appropriate set-up.

On the basis of Call’s experimental set-up and procedure, we conducted three consecutive experiments in which dogs had to find a reward that was hidden behind one of two V-shaped fences in order to test whether dogs were sensitive to the information that they themselves have or have not acquired and whether they seek extra information in situations of uncertainty. We manipulated the type (Experiment 1) and quality of reward (Experiment 2), as well as the time delay (Experiment 3) between baiting and choosing to analyze if the dogs’ searching behavior was affected.

Dogs were presented with a Seen and an Unseen condition. They could make their decision by walking around the V-shaped fence, and they could check before choosing through the corner of the V to see or smell whether the reward was there. Based on the study of Call (2010), we predicted that the dogs would check more frequently before choosing when they had not seen where the reward was baited (Unseen condition) than in cases when they had. We further predicted that dogs would show more flexibility when searching for a toy (being a concrete object they often search for) than when searching for food pieces (Experiment 1); that dogs would be more likely to check when high-quality food was hidden as opposed to low-quality food (Experiment 2); and that for higher time delays between baiting and choosing, dogs would check more or have a reduced accuracy in finding the reward (Experiment 3).

## Experiment 1: Does the type of reward impact dogs’ accuracy in an information-seeking task?

In the first experiment, we wanted to investigate if the witnessing of baiting (Seen and Unseen conditions) and the type of reward (toy or food) had an impact on dogs’ accuracy to find the baited reward. Therefore, we tested subjects in the Seen and Unseen conditions, and half of the dogs searched for food as a reward whereas the other half searched for their favorite toy. We predicted that if dogs did not know what they had seen, they would seek extra information. For the type of reward, we expected the dogs to show more flexibility when searching for a toy (which they often do in their daily life) than searching for food pieces, as the favorite toy is a concrete object that the subjects know.

### Methods

#### Subjects

In total, 48 dogs (22 males and 26 females) of various breeds and ages (range 1.5–11 years, mean 4.6 years) participated successfully in the experiment. All subjects lived as pets with their owners and received the normal obedience training typical for domestic dogs. The dog owners were not present during the test and they were informed about the precise research question as well as about the specifics of their dogs’ tasks in the study only after the completion of the test, in order to avoid potential training (by the owners).

The owners decided voluntarily to participate in this study, and if they were interested they were provided with the video material of the performance of their dog after the test was completed. All of the dogs were naïve to the information-seeking task and did not have any prior knowledge of the experiment. They were all healthy individuals with no known sight or hearing impairments and no known history of aggression towards humans. Another precondition for this experiment was that dogs had to be interested in food or toys in order to participate in this study. For the toy condition, owners were asked to bring their dogs’ favorite toy to the testing sessions. In total, 24 dogs were rewarded with food and 24 other dogs were rewarded by playing with their favorite toy. Females were not tested during estrous.

#### Materials

**Fig. 1:**
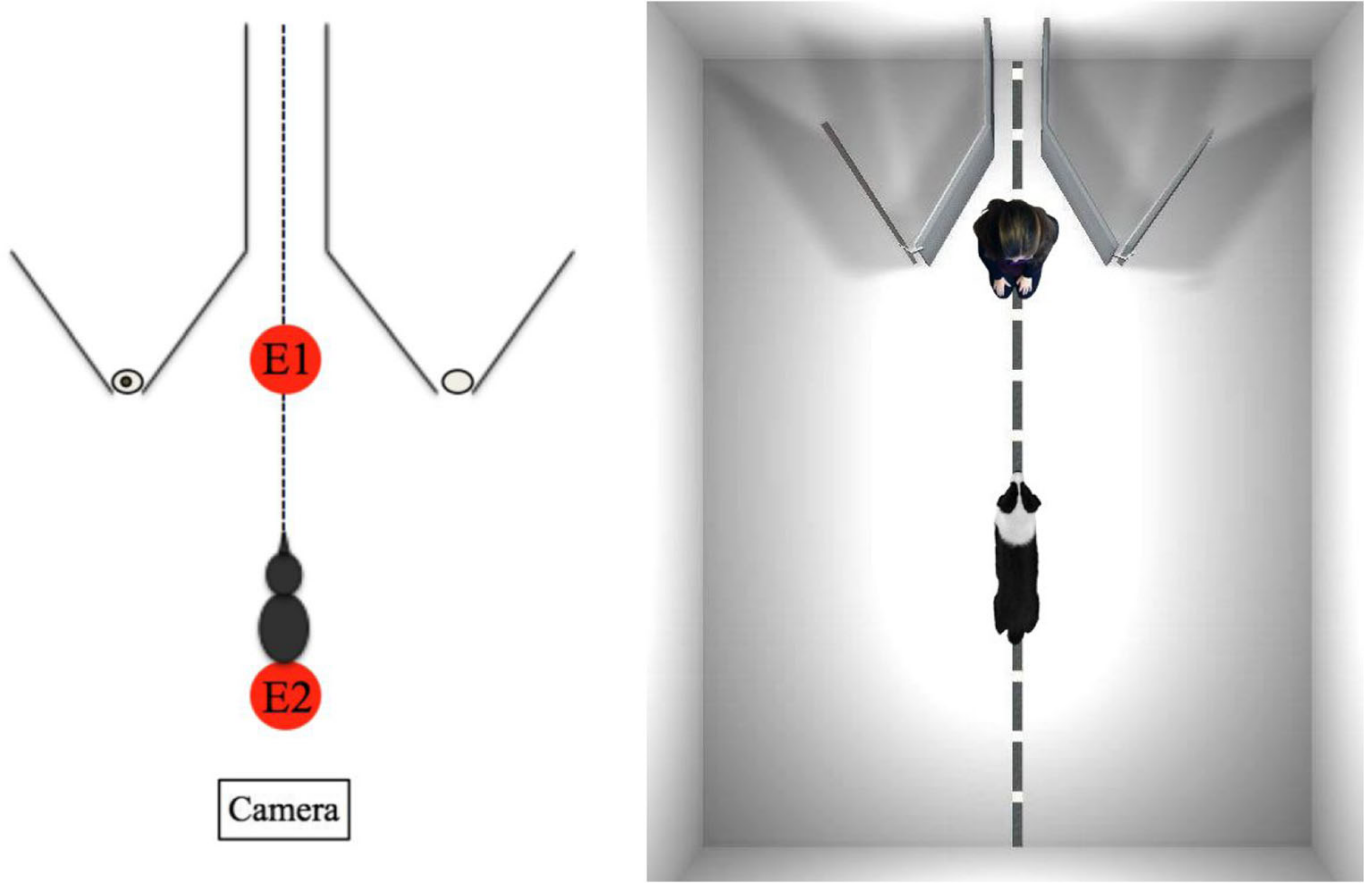
Basic set-up for Experiments 1, 2, and 3

The test took place in a quiet room (8.5 m × 4 m) at Alte Messe in Leipzig, Germany. The experimental set-up (Fig. 1) was comprised of a two-part apparatus. Each side consisted of two V-shaped wooden fence structures (1.20 m × 1.00 m) that were connected with a flexible hinge at the upper end to form a V-shape with a 45° angle. At the lower end of each V-construction was a gap of approximately 2 cm in width, through which the first experimenter (E1) placed the reward as bait on a small plate. Subjects could check whether the reward was actually hidden there or not and make their decision based on this information by walking around the fences. The distance between the corners of both barriers was 1.55 m. A centerline indicated both the exact middle of the room as well as the exact middle of the apparatus. Another marking, 1.60 m away from the corners, indicated the exact position (i.e., the nearest point) at which the dog had to wait at the beginning of each trial. E1 sat in the middle between the two fences and was responsible for baiting the reward, and the second experimenter (E2) was located next to the centerline at the starting position to hold the dog, both facing E1. The dogs had to choose one side and move around the V-shaped fences, which was only possible by walking around the outer sides. Two additional barriers prevented the subjects from passing E1 and going around the inside to fetch the reward. The dogs were rewarded with either food or their favorite toy. In the Unseen condition, a curtain was installed to prevent dogs from witnessing the baiting. All trials, including the pretest, were video-recorded by one camera that was installed directly across from the apparatus.

#### Procedure and design

All experiments in this study consisted of three consecutive units: familiarization, pretest, and experimental phase. First, each dog received a familiarization to become familiar with the testing room and to understand how to properly find the reward at the corner of the fence. Accordingly, this was followed by a pretest, which had to be successfully passed in order to make sure that all participating subjects understood the experimental set-up. Only dogs that passed the pretest took part in the experiment.

We varied whether subjects received food or their favorite toy as a reward. Therefore, one fundamental assumption was that dogs that participated in this study had either a high degree of interest in food or their favorite toy, and were furthermore motivated to fetch the reward after being released. This was confirmed in the pretest and during the familiarization sessions. As dogs have trouble obtaining a reward that is placed at the inner corner of a V-shaped fence, even if the fence is transparent (Pongracz, Miklosi, Vida, & Csanyi, 2005), we gave subjects the opportunity to investigate the testing room with the two V-shaped fences before the actual experimental phase (Pongrácz, Vida, Banhegyi, & Miklósi, 2008). Before the final experimental phase began, three dogs were tested in a pilot study. None of these subjects was included in this study. In the following section, we will explain the experimental procedure in more detail.

#### Familiarization

Before the actual test, we introduced the dogs to the testing room to ensure that they understood the apparatus and were able to find the reward without checking. The familiarization was conducted successively, meaning that the subjects became familiar with the task step-by-step. During the familiarization sessions many breaks were given. Depending on the condition, the dog was either rewarded with food or his or her favorite toy. Similar to the tests, familiarization was always conducted by the same experimenter E1, who baited the reward, and an arbitrary second experimenter E2, who held the dog at the starting position. E1 used a certain command to motivate the dogs to find the baited reward (e.g., German “Ok,” “Such!” (“Look!”), “Wo ist es?” (“Where is it?”)). When the dog approached the reward he or she was rewarded either by eating the food or by playing with the toy with E1.

At first, E1 led the way and showed the dogs where the treat was placed by walking around the fence and hiding the reward behind the corner. E1 used nonverbal cues, such as pointing, showing, and eye gaze to further assist the dog in finding the treat. After the baiting was finished, the dog was released to search for the reward. In subsequent trials E1 placed the reward through the gap. The procedure was repeated until the subject approached the reward by going around the fence without trying to get the reward through the gap.

The speed of familiarization always depended on the dog’s individual learning progress and motivation to find the reward. Dogs were given a break from familiarization either when they performed the action successfully, or when their willingness, motivation, or attention was significantly decreased.

#### Pretest

In order to pass the pretest, the dogs had to be able to walk around the fence where the reward was placed, without checking. The subjects passed the pretest when they found the reward in four consecutive trials or four out of six trials without checking through the gap. In the pretest, we did not apply any manipulation and therefore subjects witnessed the baiting procedure completely and had no delay between baiting and choosing. Only subjects that passed the pretest could take part in the actual experimental phase in the second and third sessions. However, if dogs showed no interest in participating, if they did not learn to find the food behind the fence within 120 min, or showed no interest in the reward, they were excluded from this study and marked as dropouts. For this reason, we had to exclude six dogs from the study.

#### Experimental phase

After becoming familiar with the testing room and passing the pretest, the subjects were tested in two consecutive sessions. The general procedure in the experimental trials was the same for all dogs: Two experimenters, E1 and E2, tested all subjects individually. One experimenter (E1) had to be the same person for all trials, as in the pretest. The second experimenter (E2), however, could be any person. At the beginning of each trial the dog was held by E2 at the starting position while E1 knelt between the two fences. E1 then held up the reward to show it to the dog while calling his or her name to get the dog’s attention. The baiting process differed according to two conditions:

1. In the Seen condition, E1 baited the reward while allowing the dog to see the baiting process. E1 leaned over one fence and put the reward through the gap onto the plate behind of the fence. E1 then returned to the middle of the fences, placing her arms parallel to her body.
2. In the Unseen condition, E2 closed the curtain so that the dog could not see the baiting process. E1 touched first the left and then the right gap of the two fences while placing the reward through one of them. After that, E1 again touched both gaps simultaneously in order to make sure that the subject could not hear where the reward was baited. Then she went back into the middle of the two fences, placed her arms parallel to her body, and told E2 to open the curtain.

After the baiting process was complete, E2 released the dog and E1 called his or her name and encouraged him or her to find the reward. In both conditions, E1 did not move and avoided giving any cues to the dog. She waited until the dog had made his or her choice by walking around one fence. If the dog chose the correct fence he or she was allowed to eat the food or to fetch the toy, and E1 played with him or her by throwing the toy. If the dog chose the wrong fence, i.e., where the reward was not hidden, E1 took him or her by the collar and led him or her behind the correct fence. E1 showed the reward to the dog but the dog was not allowed to eat it or play with it. After the dogs had eaten the food or played with the toy, or the reward was shown to them (when they were wrong), the trial was over and a new one began.

The reward was placed behind one of the barriers in the Seen condition only when the dog looked and paid attention to E1. The dog’s attention was essential for the continuation of the experiment as the dog needed to witness the whole baiting process. After placing the reward, E2 leaned back to the middle and placed his or her arms parallel to his or her body without looking at the dog. It was important that both E1 and E2 did not give any accidental cues (e.g., gaze, pointing, non-verbal cues) and, thus, they looked down at the floor while waiting.

Half of the dogs were tested with the food reward and half of the dogs were tested with the toy reward. They were presented in two sessions on 2 days, so that each dog received the Seen condition 12 times per day and the Unseen condition 12 times per day. Within a session, there was a break after half of the trails. The order of the conditions and the location of the food were randomized, with the stipulation that a condition occurred no more than two trials in a row, and that the food was not hidden on the same side in more than two consecutive trials. Each dog received 24 trials of each of the two conditions, totaling 48 trials (see Online Supplementary Materials for details).

#### Data scoring and analysis

All trials were analyzed from the videotapes. We scored the following three variables for each trial: success (correct choice), checking, and latency. For success we scored whether the dogs selected the correct fence, having at least the front paws and shoulder behind the outer side of the fence where the reward was baited. For checking behavior we coded whether and where the dogs checked before choosing by approaching the gap, having the mouth less than 10 cm from the gap while hesitating for at least a half a second. Finally, we scored the latency to select a fence in the trials when subjects did *not* check. Therefore, we recorded the time from E1’s first call of the dog’s name until his or her front paws and shoulder had crossed the outer part of the V-shaped fence.

To assess inter-observer reliability, one independent observer scored a randomly selected sample of 20% of the trials where the dogs were rewarded with food and the trials where the dogs were rewarded with a toy. Reliability was excellent for correct selection (food: Cohen’s Kappa=0.98, N=240; toy: Cohen’s Kappa=0.98, N=239), for checking behavior (food: Cohen’s Kappa=0.95, N=240; toy: Cohen’s Kappa=0.77, N=239), and for the latency to select (food: Pearson Correlation r=0.80, N=182; toy: Pearson Correlation r=0.77, N=153).

For the analysis, we used repeated measures 2 × 2 ANOVAS with the within-subject-factor condition (Seen vs. Unseen) and the between-subject-factor reward (food vs. toy). To test for learning over trials, we used repeated measures 2 × 2 × 2 ANOVAS with the within-subject-factors condition (Seen vs. Unseen) and session (first vs. second session) and the between-subject-factor reward (food vs. toy). For comparisons against chance within one condition, one-sample t-tests were used, as indicated.

### Results

#### Success

The dogs selected the correct fence in 94% of the trials in the Seen condition and in 57% of the trials in the Unseen conditio,n and were above chance in both conditions (Seen: t(47)=31.09, p<0.001; Unseen: t(47)=3.90, p<0.001, one-sample t-tests). They performed better in the Seen than in the Unseen condition (F(1.46)=282.04, p<0.001), and they showed increased accuracy when they were rewarded with the toy (F(1.46)=5.95, p=0.019), but there was no interaction between Condition × Reward (F(1.46)=1.77, p=0.190).

#### Checking

**Fig. 2:**
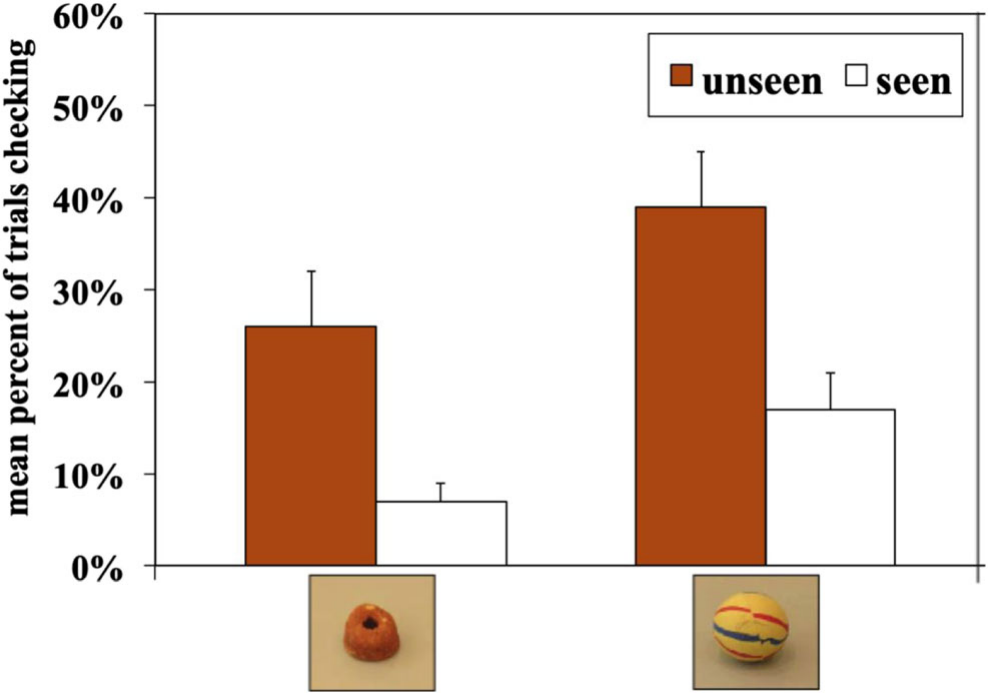
Mean percent of trials in which the dogs checked for the different rewards in the conditions (+/-SE) in Experiment 1

Figure 2 presents the mean percentage of trials in which the dogs checked for the different rewards in the two conditions. The dogs checked more frequently in the Unseen condition than in the Seen condition (F(1.46)=35.69, p<0.001), and they tended to check more when they were rewarded with the toy (F(1.46)=3.91, p=0.054). There was no interaction of Condition × Reward (F(1.46)=0.28, p=0.601).

#### Checking and success

**Fig. 3:**
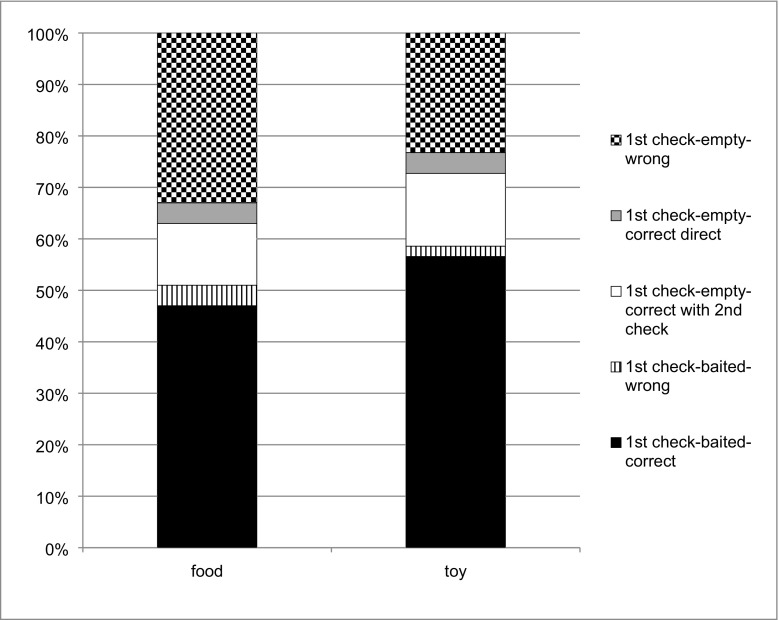
Percentage of dogs’ performance regarding checking behavior followed by their decision for one side depicted for food and toy in the Unseen condition in Experiment 1. Depicted are all five possibilities of dogs’ checking behavior with food and toy rewards, i.e., which fence they attempted first when they check and which side they selected. By chance dogs can first check the baited side, then they can either go to the correct side (first check-baited-correct) or – wrongly – to the fence where the reward is not hidden (first check-baited-wrong). When dogs check the wrong side on their first attempt, they can either then select the wrong side (first check-empty-wrong), or they can do a second check at the baited side and select the correct fence (first check-empty-correct with second check), or they can choose the baited side without further checking (first check-empty-correct direct)

If subjects checked in the Seen condition, they then selected the correct fence above chance in 95% of the cases (t(37)=20.32, p<0.001, one-sample t-test). Similarly, if they checked in the Unseen condition they were correct above chance in 68% of the cases (t(44)=4.41, p<0.001, one-sample t-test). Thus, the dogs’ success rate was higher when they checked in the Seen condition than in the Unseen condition (F(1.35)=40.47, p<0.001). There was no effect of reward (F(1.35)=2.65, p=0.112) and there was no interaction between Condition × Reward (F(1.35)=1.47, p=0.234). Figure 3 illustrates the checking behavior of the two groups of dogs in the Unseen condition.

#### Latency

Food-rewarded dogs selected a fence faster than toy-rewarded dogs in the cases when they did not check (F(1.45)=12.20, p=0.001), but there was no effect for condition (F(1.45)=2.18, p=0.147) and no interaction effect (F(1.45)=0.06, p=0.816).

#### Learning

Regarding success, we found no learning over trials in this experiment. Subjects did not select the correct fence more often in the second session compared to the first session (F(1.46)=0.008, p=0.930). However, there was a significant interaction effect (Condition × session × reward: F(1.46)=4.429, p=0.041). In contrast, subjects checked more in the first session than in the second session. There was a significant effect for session (F(1.46)=8.099, p=0.007), but no interaction effect.

#### Individual performance

Individuals were above chance when they selected the correct fence in 18 (75%) out of 24 trials or more (binomial test p=0.5, N=24, P=0.02). Forty-five dogs were above chance in the Seen condition (21 food rewarded and all 24 toy rewarded dogs). Eight dogs were above chance in the Unseen condition (two food-rewarded and six toy-rewarded dogs). Two food-rewarded dogs never checked whereas all toy-rewarded dogs checked at least twice.

### Discussion

The dogs checked more often before selecting the correct fence when they did not see where the reward was hidden. They showed a flexible checking behavior, indicating that dogs may have access to their own visual perception. Similar to primates (Call, 2005; Call & Carpenter, 2001; Hampton et al., 2004; Marsh & MacDonald, 2012; Perdue, Evans, & Beran, 2018), they sought extra information when they did not know the reward’s location. When dogs did not witness the baiting, they were able to adapt their behavior by gathering additional information that might have led to success; by checking, they could select the correct fence where the reward was hidden. Moreover, they were able to revise their choice when they began their inspection at the wrong fence.

Overall, the dogs showed similar checking and searching patterns to primates. However, there were three differences compared to primates.

First, dogs in general checked less than the apes tested by Call (2010) and Call and Carpenter (2001), and were therefore less likely to be successful in the Unseen condition (they only performed slightly above chance level). Second, having begun checking the contents of the containers before choosing, the apes continued to do so throughout the remaining trials (Call & Carpenter, 2001). For the dogs, we did not find such an effect. Dogs either checked very often or rarely. Moreover, there was a decrease in checking behavior between the first session and the second session. Thus, although the dogs checked less in the second session, they did not learn the most effective strategy over trials, i.e., checking more when they had not seen and less when they had seen where the reward was hidden. This means that their flexible checking behavior was not learned during the experiment.

The third difference was that the dogs – in contrast to the apes – were not always accurate when they checked. This might be due to the set-up, as looking and smelling through the narrow gap might lead to less accuracy than looking inside a tube. But still dogs as a group were able select the correct fence above chance level in the Unseen condition.

Can we conclude from these results that dogs have access to what they have seen? Studies using the information-seeking paradigm have been criticized because subjects may simply engage in a search for information routinely without any metacognitive involvement. According to this hypothesis, individuals engage in a variety of exploratory responses until they detect the reward (Hampton et al., 2004; Kornell et al., 2007).

However, as Call (2010) pointed out, this is unlikely for two reasons: the tested primates selected the correct tube in about 20% of the trials after only having looked inside the empty tube (Call & Carpenter, 2001; Call, 2005; Marsh & MacDonald, 2012; Perdue, Evans, & Beran, 2018). Dogs in the current study were also able to make this inference by exclusion (as was also shown in other studies, see, e.g., Aust et al., 2008; Erdohegyi et al., 2007; Wallis et al., 2016), although less than the primates, in about 5% of the cases. This means that subjects did not need to smell or see the reward to select the correct alternative. The second reason why it is unlikely that subjects simply engaged in a search for information routinely is that the dogs, like the primates, also checked when they had seen where the reward was hidden (in more than 10% of the trials). However, it is unlikely that they had forgotten the location of the reward because the delays were very short and subjects were correct in nearly 100% of the trials even when they did not check.

Our results are in contrast to previous findings of Bräuer et al. (2004) and McMahon et al. (2010), Experiment 1. The dogs in these studies were apparently influenced by the fact that they were trained before the test in how to select the correct box. In the current study the dogs also had some previous experience with the apparatus but they did not have to learn to press a lever or a cue to locate the reward. Thus, with the current paradigm dogs could search naturally for the reward and it was shown that they are able to distinguish between a situation in which they had and had not seen where the reward was hidden. Our results are supported by Experiments 2 and 3 of McMahon et al. (2010), in which dogs had a choice between an informant and a non-informant. The dogs preferred to approach the informative human who then pointed to the location of the reward. This again suggests that dogs are seeking extra information when they do not know where the reward is hidden.

Interestingly, dogs selected the correct fence more often when they were rewarded with the toy, and they then also tended to check more often. Thus, it is possible that dogs search in a more flexible way when they are rewarded with a toy. Dogs also showed flexible searching strategies when they searched for a toy in a number of other studies (Erdohegyi et al., 2007; Fiset, 2009; Fiset et al., 2000, 2003, 2006). Why were dogs more successful with the toy? One possibility is that they were able to perceive the toy better when they were checking through the gap, as it is bigger. The second possibility is that the dogs rewarded with food were less motivated to search for their reward than the dogs that searched for the toy. That is very unlikely because the dogs actually approached the food reward even faster than the toy reward. It could, however, be the case that the dogs were too motivated to get the food reward, so that it was more difficult to be patient enough to check before choosing. In other words, dogs may have been more impulsive, and therefore less likely to show metacognitive abilities, when the reward was food. A third possibility is that the dogs perceived the two rewards in different modalities. It is not clear how the dogs perceived the reward behind the gap, whether they saw or whether they smelled it. It is possible that the dogs used smell to check for the food and vision to check for the toy, and that the visual modality makes them more flexible (see also Szetei et al., 2003). In an information-seeking experiment with capuchin monkeys conducted by Vining and Marsh (2015), subjects were either shown where the food was hidden, they could infer its location, or they were not given information about the location of the food. Monkeys also had the opportunity to search for extra information, and similar to our dogs they used this opportunity especially in the Unseen condition but less in the Seen condition. But when the monkeys potentially could infer the reward’s location, they were more likely to search for further information. The authors conclude that capuchins only metacognitively control their information seeking in situations in which information is presented in the visual domain (Vining & Marsh, 2015).

The fourth possibility lies in the nature of the rewards. The favorite toy is a concrete object that the subjects know. Thus, it is a focused search, as subjects know exactly what they are looking for. In contrast, searching for food is more diffuse, as there could potentially be more pieces around (although subjects probably perceived that the test is about one piece). Moreover, the dogs in their daily life probably have much more experience with searching for a toy, and especially their favorite toy, than searching for food.

## Experiment 2: Do subjects check more when a high-quality reward is involved?

In Experiment 1, we demonstrated that dogs seek out extra information when they have not seen where a reward was hidden and that they were more accurate when their favorite toy was hidden. Following Call (2010), we were interested in the question whether the location of a high-quality reward was better remembered than the location of a low-quality reward. We predicted that dogs would check more often when a high-quality reward was baited as opposed to a low-quality reward. In this experiment, new subjects that were unfamiliar with the task were presented with two types of reward in Seen and Unseen trials.

### Methods

#### Subjects

We tested 24 dogs that did not take part in the previous experiment but were chosen based on the same selection criteria. As food was given as a reward, it was crucial for the experiment that dogs were motivated by food. There were 12 females and 12 males ranging from 1 to 6 years of age. As in Experiment 1, all subjects lived as normal family dogs and were individually tested at the Alte Messe in Leipzig, Germany.

#### Materials

The same experimental set-up was used, including the apparatus with the aforementioned two V-shaped wooden fence structures as in Experiment 1. Again, a gap of approximately 2 cm in width was used to hide the reward. For high-quality food, dogs were given meat sausages (“Hundewürstchen”), while dry dog food served as low-quality food.

#### Procedure and design

The basic procedure was the same as in Experiment 1: While E1 kneeled between the two barriers facing the dog, E2 held him or her at the starting position. The two experimenters tested all subjects individually, whereby one experimenter (E1) placed a piece of food behind one of the fences, as was the procedure in Experiment 1.

After the familiarization and the pretest, a classical food preference test was conducted before each session to ensure that dogs had a preference for one type of food. We assumed that dogs would prefer meat sausages as high-quality food as opposed to dry dog food, which was seen as low-quality food. The preference test was conducted in another part of the room, where dogs were presented with a wooden table. To ensure that dogs really preferred the meat sausages (the high-quality food) over the low-quality reward, E1 sat behind the table across from the dog and fed him or her with a piece of high-quality and a piece of low-quality food. E1 moved towards the dog, holding a piece of food in each hand close to the dog’s nose, and then placed simultaneously a piece of each type of food at the end of the board. Dogs were included if they showed a clear preference for the high-quality reward (sausages), i.e., chose the high-quality reward above chance level in the four food preference tests. Overall, dogs chose the preferred food in 89% of trials (t(23)=16.31, p<0.001, one-sample t-test).

Only if the pretest and food preference test were successfully passed, were the Seen and Unseen conditions, which were similar to Experiment 1, introduced. It was also varied whether high-quality food (sausage) or low-qualify food (dry food) was hidden, resulting in four conditions: Seen-high / Seen-low / Unseen-high / Unseen-low. Dogs were tested in four sessions (two sessions per day). Each session consisted of 16 trials: the food preference test has four trials and each of the four main conditions contained three trials. All trials were presented randomly with the requirement that a condition occurred in no more than two trials in a row. Moreover, the food was not hidden on the same side in more than two consecutive trials.^1^

#### Data scoring and analysis

All trials were videotaped and scored in the same way as in Experiment 1. Thus, we scored success (correct choice), checking, and latency. The inter-rater reliability, which was based on 20% of the trials, was very good for correct selection (Cohen’s Kappa=0.988, N=24), for checking behavior (Cohen’s Kappa=0.792, N=24), and for the latency to select (Pearson Correlation r=0.936, N=24). For the main analysis we used a 2 × 2 ANOVA with the within-subject factor condition (Seen vs. Unseen) and the between-subject factor reward (high- vs. low-quality reward).

### Results

#### Success

The dogs selected the correct fence in 94% of the trials in the Seen condition and in 52% of the trials in the Unseen condition, and were above chance in the Seen condition (t(23)=22.20, p<0.001), but not in the Unseen condition (t(23)=1.12, p=0.274, one-sample t-tests). They were more accurate in the Seen than in the Unseen condition (F(1.23)=239.74, p<0.001), but there was no effect for the type of food (F(1.23)=0.64, p=0.429), and no interaction effect (F(1.23)=0.89, p=0.354).

#### Checking

**Fig. 4:**
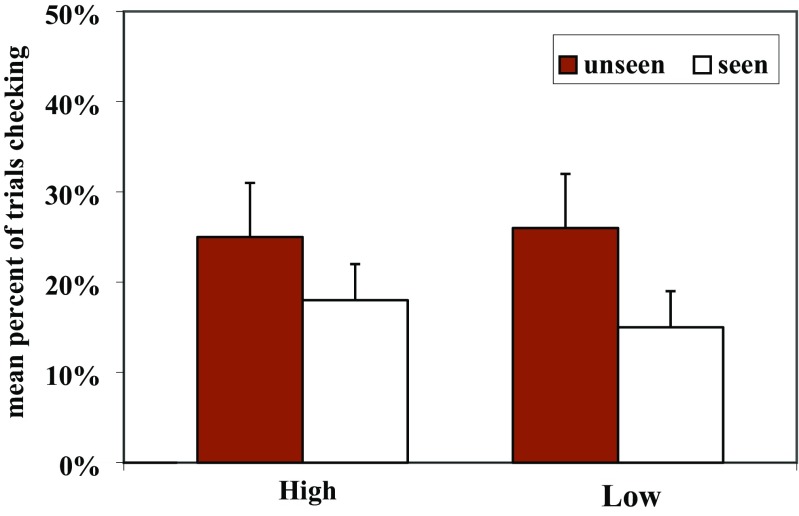
The mean percentage of trials in which dogs checked for the two different food rewards in the two conditions in Experiment 2

Figure 4 presents the mean percentage of trials in which the dogs checked for the two different food rewards in the two conditions. The dogs checked more frequently in the Unseen condition than in the Seen condition (F(1.23)=8.32, p=0.008), but there was no effect for the type of food (F(1.23)=0.74, p=0.400) and no interaction effect (F(1.23)=1.00, p=0.328).

#### Checking and success

If subjects checked in the Seen condition they then selected the correct fence above chance in 98% of the cases (t(18)=23.82, p<0.001, one-sample t-test). However, if they checked in the Unseen condition they were correct only in 60% of the cases, which was not above chance (t(21)=1.44, p=0.165, one-sample t-test). The dogs’ success rate was higher when they checked in the Seen condition than in the Unseen condition (F(1.9)=18.76, p=0.002). These results suggest that dogs in the Seen condition simply might have re-assured themselves that the food was still there, but did not use checking successfully in the Unseen condition. Moreover, there was no effect of type of food (F(1.9)=1.01, p=0.342) and there was no interaction between Condition × Type of food (F(1.9)=0.91, p=0.365).

#### Latency

On average it took subjects 2.8 s to select a fence in the cases when they did not check before choosing. Dogs selected a baited fence faster when the food was preferred than when it was not preferred (F(1.22)=6.59, p=0.018), but there was no effect for condition (F(1.22)=0.69, p=0.414) and no interaction effect (F(1.22)=0.20, p=0.659).

#### Learning

There was no learning over trials. Subjects did not select the correct fence more often in the second session compared to the first session: although there was a significant effect for condition (F(1.23)=239,60, p<0.001), there was no interaction effect (Condition × Session F(1.23)=2.38, p=0.137) and for session (F(1.23)=0.03, p=0.863). Similarly, subjects did not check more in the first session compared to the second session (F(1.23)=0.27, p=0.608), and there was no interaction effect (Condition × Session F(1.23)=1.28, p=0.270), but again there was an effect for condition (F(1.23)=8.30, p=0.008).

#### Individual performance

Again, individuals were above chance when they selected the correct fence in 18 (75%) out of 24 trials or more (binomial test p=0.5, N=24, P=0.02). Whereas 23 dogs were above chance in the Seen condition, only one dog was above chance in the Unseen condition. Two dogs never checked at all, 22 dogs checked at least once in the Unseen condition and 19 dogs checked at least once in the Seen condition.

### Discussion

As in Experiment 1, dogs checked more frequently in the Unseen condition than in the Seen condition, but there was no effect for type of reward. Dogs remembered the locations of both food types equally well, and did not remember the location of a high-quality reward better. We predicted that dogs would check more often when a high-quality reward was baited as opposed to a low-quality reward, independent of whether subjects had or had not witnessed the baiting. However, that was not the case as dogs showed the same patterns for checking, no matter whether the food was preferred or not.

One could argue that dogs did not perceive or forgot which type of food was hidden. However, dogs selected the baited fence faster when a high-quality reward was hidden compared to a low-quality reward, indicating that they did indeed know which food was hidden. This increased selection of preferred food could indicate some evidence for the response competition hypothesis (Hampton, Zivin, & Murray, 2004). It predicts that if a higher value reward is available, the subject will be more motivated to go for it, i.e., in our case to go faster. However, as we did not find *decreased* checking for the high-value reward, the evidence remains weak.

In sum, in contrast to apes and humans, dogs’ checking response was independent of the value of the reward, although they were aware of the type of food that was hidden.

## Experiment 3: Does forgetting predict checking?

In this experiment, we raised the question whether time delay had an impact on dogs’ accuracy and checking responses. The delay between baiting the reward and selecting one of the fences was manipulated to foster forgetting and examine whether checking would increase accordingly. Thus, we adapted the previously used information-seeking paradigm and varied the time delay (5, 20, 60, 120 s) between baiting the fences and letting dogs choose one side (similar to Call, 2010). Longer time delays are associated with a higher degree of difficulty to locate a baited reward (Call, 2010). Because forgetting would predict an increase in checking, we proposed that longer time delays lead to greater forgetting and, thus, foster checking.

### Methods

#### Subjects

The selection criteria for the subjects were the same as in the previous two experiments, i.e., dogs had to be interested in food and to be able to pass the pretest. All subjects were normal family dogs that lived as pets with their owners in Jena and surroundings. In total, 25 privately owned dogs (11 males and 14 females; mean age = 5.21 years) of various breeds and ages (range 1–12 years) participated for the first time in this kind of experiment. All 25 dogs were rewarded with food – either Frolic or, in case of food allergies, equally preferred food.

#### Materials

All tests were conducted in a test room (7.20 m × 5.50 m) at the Dog Lab of the Max Planck Institute for the Science of Human History in Jena from April to August 2017. The experimental set-up was exactly the same as in the previous experiments. Additionally, we used two thick blue mats that were placed in front of the gaps and served to block visual access to the fences’ contents (see below). All trials, including the pretest, were video-recorded by one camera that was installed directly across from the apparatus.

#### Procedure and design

In this experiment, the general procedure was the same as in the Seen condition of Experiments 1 and 2, but we varied the time delay between baiting and the dog’s release. We used time delays of 5, 20, 60, and 120 s. E1 measured the exact time delays with a stopwatch, starting right after the reward was baited and E1 was in the initial position.

Similar to Call (2010), we also wanted to implement a “Blocked” condition in which dogs were prevented from checking by placing two thick blue mats in front of the gaps. However, as dogs did not show any difference in their behavior between the conditions “Blocked” and Unblocked” for success, checking, or latency, we concluded that we could not prevent them from checking as they used their nose to check. Thus, as this manipulation did not work, we treat the “Blocked” and “Unblocked” trials as one condition.

In total, each subject received two administered 24-trial blocks (one block for Seen and one block for Unseen trials), resulting in a total of 48 trials. The order of the time delays (5, 20, 60, and 120 s) was randomized for all dogs within eight trials and repeated in the exact same order afterwards. Food was placed an equal number of times on each side with the only restriction that the reward was hidden not more than twice in a row in the same place in a session.

#### Data scoring and analysis

All trials were analyzed and scored from the video material in the same way as in Experiments 1 and 2. Thus, we used the three measures checking, success, and latency.

In order to assess inter-rater reliability, a second observer unfamiliar with the task scored a randomly selected sample of 20% of the trials, which equaled a total of five dogs. Subjects were chosen randomly. For all measures, the inter-rater reliability was excellent and similar to Experiments 1 and 2 (Correct choice: kappa = 1.0, Checking: kappa = 0.80, Latency: Pearson Correlation r=0.78, N=25). For the main analysis we used a 1 × 4 ANOVA with the within-subject factor time delay.

### Results

#### Success

**Fig. 5:**
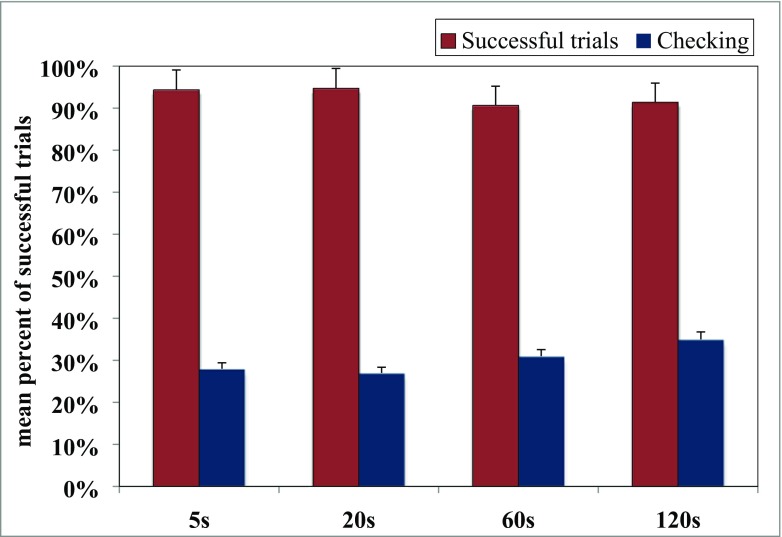
The mean percent of trials in which dogs retrieved the reward successfully in Experiment 3. Dogs showed higher accuracy in trials with shorter time delays compared to trials with longer delays between baiting and choosing

On average, dogs selected the correct fence in 93% of the trials.^2^ For a time delay of 5 s, they chose the correct side in 94% of trials, for 20 s in 95% of trials, and for both 60 and 120 s in 91% of trials (see Fig. 5). We found a significant effect for time delay (F(3.72)=3.21, p=0.028). A paired-sample t-test revealed that dogs were significantly more accurate in 5 s compared to 60 s (t(24)=2.681, p=0.013), 20 s compared to 60 s (t(24)=2.071, p=0.049, and 20 s compared to 120 s (t(24)=2.089, p=0.047).

#### Checking

On average, dogs checked in 28% of trials with a time delay of 5 s, in 27% of 20 s, in 31% of 60 s, and in 35% of 120 s (see Fig. 5). We had assumed that dogs would check more when the task is more difficult, e.g., when the time delay between hiding and the possibility of searching for the food is longer. However, the statistical analysis revealed that there were no significant differences between the four time delays. Dogs did not check more often depending on time delay (F(3.72)=2.086, p=0.11).

#### Latency

Subjects took on average 3.1 s to select a fence in the cases when they did not check. There was no effect for latency (F(3.69)=2.038, p=0.12), thus, subjects did not take significantly longer to retrieve the reward as a function of delay when they did not check.

#### Learning

In this experiment, there was no learning over trials. Subjects did not select the correct fence more often in the last session compared to the first session: Although there was a significant effect for delay (F(3.72)=3.21, p=0.028), there was no interaction effect (delay × session F(3.72)=1.21, p=0.31) and for session (F(1.24)=1.81, p=0.19). Similarly, subjects did not check less in the first session as opposed to the second session (F(1.23)=0.104, p=0.75), there was no interaction effect (delay × session F(3.69)=0.79, p=0.5), and no effect for delay (F(3.69)=1.97, p=0.13).

### Discussion

In this experiment, we investigated metacognition in dogs by assessing the impact of time delay in an information-seeking task. We found that the dogs’ overall retrieval accuracy was significantly higher for shorter time delays, i.e., dogs were less accurate when the delay was longer. However, in contrast to the apes tested by Call (2010), dogs did not check more often in situations in which the task was more difficult. Thus, dogs did not search for extra information when they were uncertain, which might suggest that they did not have access to their own knowledge in that situation.

Similar to Call (2010), we also wanted to implement a “Blocked” condition in which dogs were prevented from checking by using an occlude in front of the gap. However, that manipulation did not work, as dogs did not show any difference in their behavior between the “Blocked” and “Unblocked” trials. Thus, dogs checked and were equally successful in “Blocked” trials, meaning that they were able to check through the occlude. This indicates that dogs used their olfactory sense to check whether the reward was present or absent, which is not so surprising as dogs very much rely on their nose when they search for a reward (Gazit & Terkel, 2003; Miklosi, 2007; see also Bräuer & Belger, 2018).

### General discussion

Similar to apes, monkeys, and 2.5-year-old children (Call & Carpenter, 2001; Hampton et al., 2004), dogs tend to actively seek extra information when they have not seen where a reward is hidden. Although subjects checked more often before selecting the correct fence when they did not see where the reward was hidden (Experiments 1 and 2), their searching behavior was not affected by their preference for a type of food (Experiment 2). Manipulating the time delay between baiting and choosing slightly affected dogs’ performance: subjects were significantly less accurate, but they did not check more often for higher time delays (Experiment 3). In contrast to previous studies (Bräuer et al., 2004) we were able to demonstrate that dogs showed some aspects of information-seeking behavior related to metacognition, but less flexibly than apes.

The main objective was to examine whether dogs were sensitive to the information that they themselves have or have not seen and whether they seek extra information in situations of uncertainty. As shown in Experiments 1 and 2, dogs checked more when they did not witness the baiting procedure. This suggests that they expected the bait was hidden behind one of the fences and, additionally, they grasped that they did not have enough information about where exactly it was hidden. Therefore, dogs must have adapted their searching behavior to increase their chance of success. We showed that dogs checked more in the Unseen condition, although they were not as successful as in the Seen condition when they checked. We were able to replicate this effect in two experiments with two independent cohorts of dogs.

However, we found one major difference between Experiment 1 and Experiment 2 regarding the performance of the two cohorts of dogs (probably due to the fact that a toy reward was easy to follow, see discussion of Experiment 1 and below). While checking in the Unseen condition in Experiment 1 indeed helped dogs to increase their accuracy above chance level, increased checking behavior in Experiment 2 did not lead to a higher accuracy. In other words, although dogs checked more often in the situation of uncertainty (i.e., when they did not witness the baiting process) in Experiment 2, they did not find the food more often than what was expected by chance. Thus, although this did not lead necessarily to increased success, dogs looked for extra information. They sometimes checked, but not until they were certain where the food actually was. This might indicate that dogs sometimes have a problem inhibiting the approach to the reward, even when they perceive that they need to gather extra information. Apes in the study of Call (2010) did not have that problem. They could get the information with a glance into the tube. However, overall, dogs were potentially able to gather enough information through the gap in order to get enough information to find the reward (as proven in Experiment 1).

All dogs that passed the pretest indicated that they understood the experimental set-up and they also knew that a reward was hidden behind one of the fences. Similar to the tip-of-the-tongue phenomenon – I know that I know something but cannot retrieve the information – checking in the Seen condition could be seen as some kind of verification process to maximize the chance of reward. The fact that dogs checked more when they had no knowledge of the reward’s location (Unseen condition) could suggest that dogs show metacognitive abilities, as they meet one of the assumptions of knowing about knowing (Beran, Brandl, Perner, & Proust, 2012; Fleming, Dolan, & Frith, 2012; Hofer & Pintrich, 1997; Metcalfe & Shimamura, 1994; Nelson & Narens, 1994).

From our results we can furthermore conclude that dogs not only showed increased accuracy but also checked more when the reward was their favorite toy as opposed to food. One possible reason for this is that the dogs’ motivation was higher when a toy was at stake (see also above). Another possibility is that they smelled the food and therefore checked less often. However, dogs were faster in approaching food than approaching the toy behind the fence (when not checking), suggesting that their motivation for food was higher. Hence, one might argue that dogs had an inhibition problem for food, which is furthermore confirmed by the food quality condition: dogs retrieved the preferred food faster than the less preferred food, although there were no effects for success as well as for checking. Thus, we speculate that the latency to approach the food might be correlated with motivation. The higher the motivation, the less dogs are able to inhibit a direct approach without checking. Consequently, dogs’ greater performance with a toy reward might not only be explained by their experience with searching for a toy but also by the fact that they are not as over-motivated as with food. Indeed, in an inhibition task with food, dogs were shown to commit a number of seek errors, simply induced by ostensive-communicative cues (Topál et al., 2009).

As pointed out by Hampton (2009), several studies on non-human animal metacognition showed that difficult trials in memory or perception tests were avoided while the searching behavior could be adapted by gathering more information to maximize the reward. The dog’s overall performance may be the result of response competition theory (Hampton, Zivin, & Murray, 2004), as an alternative explanation. Knowing the location of the food may have predisposed the dogs to select a side while excluding all other options, such as searching for the reward. According to this interpretation, dogs had two competing options in our experimental design: retrieving food or searching for further information. In the Seen trials the drive to retrieve the reward was dominant, and so the dogs went directly to the location where the reward was hidden. In Unseen trials, however, the dogs did not know where the food was located and therefore the drive to search for information was more dominant (Hampton, 2009). Thus, one could even argue that searching for the food is the default behavior of foraging dogs, and this default behavior is inhibited by knowing where food is.

While Call (2010) defined the looking responses (i.e., bending down to look into the tubes) as crucial features for seeking additional information for the apes, we introduced *checking* through the gap of the V-shaped fence as an equivalent measure. Dogs did not show any differences in their performance between situations in which the gap was and was not blocked by an occlude. That means that they were able to successfully check through the occlude, indicating that they mainly used their olfactory sense to check whether the reward was present or absent. This is not as surprising as dogs very much rely on their nose when they search for a reward (see Bräuer & Belger, 2018), and their olfactory perception is proven to be excellent (Vonk & Leete, 2017). Thus, it is likely that dogs and apes used different senses for checking. Indeed, other studies have also shown that apes and dogs use different strategies to deal with the same task. For example, Bräuer and Call (2011) investigated object individuation in dogs and apes by implementing a classical violation-of-expectation paradigm. Their findings revealed that while apes showed increased begging and looking behaviors, dogs showed increased smelling when their expectation was violated (Bräuer & Call, 2011). Moreover, other studies have shown that dogs sniff more with increasing difficulty of the task, be it when searching for a toy (Bräuer & Belger, 2018) or in object permanence tasks (Gagnon & Dore, 1992), thus gathering information from other sensory modalities when one was not sufficient. Future studies investigating metacognition in dogs should therefore consider that dogs will mainly use their sense of smell when searching for extra information in situations of uncertainty.

So far, our results have only been interpreted in the light of humanlike metacognitive abilities while other alternative non-metacognitive explanations could also apply. According to the non-metacognitive anxiety model by Carruthers (2008), the subjects react to their anxiety produced by their knowledge states and not to their knowledge states, which are opaque to the individual. This alternative explanation could also offer an alternative explanation for the passport effect (Call & Carpenter, 2001; Call, 2010). Subsequently, this would imply that not receiving the high-quality reward generates a higher state of anxiety as opposed to not receiving the low-quality reward. Therefore, dogs may be more likely to seek information even though they already know where the reward is baited, since the costs of failing to locate the high-quality reward would be higher. The same anxiety model can be applied to our Seen and Unseen condition in which dogs checked more often when they had not seen where the reward was hidden: while in the Unseen condition more anxiety should result in an increase in checking, less anxiety entails less checking in the Seen condition. According to the response competition hypothesis (Hampton, Zivin, & Murray, 2004), which potentially explains behavior without evoking a metacognitive decision, checking in the Seen condition should, contrary to our results, be reduced for high-quality rewards, because the strength of the motivation to reach the food would be much higher. This, however, does not match with our results, which show that dogs checked more in the Unseen condition.

In contrast to Call’s (2010) study with apes, we did not find evidence that the dogs’ searching for extra information depended on the value of the food reward (i.e., food quality) and the time delay between hiding and searching. As for the time delays, we found that although dogs’ accuracy was better for shorter delays, they did not adapt their searching strategy to compensate their lack of knowledge by checking. In contrast to the apes, dogs checked in fewer trials, and more importantly they did not check more for longer time delays. One could argue that this was due to a ceiling effect, as dogs overall selected the correct fence in 93% of trials, and the pressure for seeking extra information was low. However, apes showed a similar accuracy (see Call, 2010, Fig. 3) but showed increased looking for longer delays. It might, however, be that dogs would show increased checking when the pressure is higher, i.e., when their accuracy gets much lower as the delays are longer. However, from the current data we can conclude that dogs do not have the flexibility that is described in the passport effect, and thus their search for extra information does not depend on the value of the reward or on the time delay between hiding and searching.

In sum, we tested in three experiments whether dogs know that they could be wrong. Our hypotheses that dogs show flexible metacognitive skills were not fully confirmed and our results were only partly consistent with Call’s (2010) results. Dogs checked significantly more in the Seen than in the Unseen condition, indicating that they may have metacognitive abilities to some extent. Checking was voluntarily used to reduce the probability of being wrong and to maximize the possible reward. However, dogs’ searching behavior for extra information did not depend on the value of the food reward or the time delay between hiding and seeking, which according to Call (2010) would be clear evidence that they knew that they could be wrong. Dogs are able to adapt their searching behavior by looking for extra information in a flexible way, indicating that they have access to what they have seen. However, further work is needed to determine which specific monitoring processes related to metacognition are involved.

## Electronic supplementary material

ESM 1 (PDF 1360 kb)

## Footnotes

1 Similar to Call (2011), we implemented 12 trials of a control condition at the end of each test to make sure that the dogs remembered where the food was hidden. None of the tested dogs made a mistake.

2 As mentioned above we did not find any significant difference between blocked and unblocked trials, and therefore analyzed the data regardless of this manipulation.
