# Supplementary material for: Metacognition in dogs: Do dogs know they could be wrong?
Source: Learn Behav. 2018 Nov 12;46(4):398–413. doi: 10.3758/s13420-018-0367-5 (PMC6276073; doi:10.3758/s13420-018-0367-5)
Supplement: Supplementary file 1 — (PDF 1360 kb) [file 13420_2018_367_MOESM1_ESM.pdf]

Supplemental tables

Table 1. List of a total of 97 dogs that participated in one of the three experiments, indicating breed, gender, experiment, and age at time of testing.

**Name**	**Breed**	**Gender**	**Age (years)**	**Exp**	**Rewarded with**
Paul	Tibet Terrier	Male	8	1	food
Jimmy	Labrador	Male	7	1	food
Balou	Flat coated Retriever	Male	4	1	food
Balou	Mongrel (Riesenschnauzer)	Male	6	1	food
Eloy	Riesenschnauzer	Male	2	1	Food
Cheyenne	Malinois	Female	3	1	food
Bonny	American Staffordshire Terrier	Female	8	1	food
Leo	Mongrel (Australian Shepherd x Border Collie)	Male	6	1	food
Jenobi	Collie	Female	3	1	food
Lotte	Mongrel (Labrador)	Female	7	1	food
Nicky	Malinois	Female	1.5	1	food
Wanja	Mongrel	Female	11	1	food
Rocky	Mongrel (American Staffordshire Terrier x Boxer)	Male	7	1	food
Linus	Golden Retriever	Male	4	1	food
Gonzo	Labrador	Male	3	1	food
Bootsmann	Labrador	Male	6	1	food
Punk	Mongrel (German Shepherd x	Male	7	1	food

	Labrador)				
Bono	Pudel	Male	2	1	food
Polly	Mongrel (German Shepherd)	Female	8	1	food
Enna	Airdale Terrier	Female	3	1	food
Aura	Riesenschnauzer	Female	3	1	food
Gina	Mongrel (Labrador)	Female	4	1	food
CJ	Border Collie	Female	4	1	food
Wilma	German Shepherd	Female	5	1	food
Arkos	Appenzeller Sennenhund	Male	1.5	1	toy
Marco	Mongrel (German Shepherd)	Male	4	1	toy
Ronja	Mongrel (Jack Russel Terrier)	Female	4	1	toy
Rudi	Airdale Terrier	Male	3	1	toy
Max	Airdale Terrier	Male	8	1	toy
Sydney	Border Collie	Male	2	1	toy
Balou	Mongrel (Border Collie x Labrador)	Male	5	1	toy
Cherry	Mongrel	Female	5	1	toy
Dusty	Border Collie	Male	2	1	toy
Pauline	Portugiesischer Waserhund	Female	5	1	toy
Karah	Labrador Retriever	Female	2	1	toy
Emily	Labrador Retriever	Female	2	1	toy
Fanny	Mongrel (Rottweiler)	Female	9	1	toy
Asta	Fox Terrier	Female	4	1	toy
Lea	Mongrel (Rottweiler x German Shepherd)	Female	7	1	toy

Luna	Mongrel (Dackel)	Female	1.5	1	toy
Bones	German Shepherd	Male	6	1	toy
Fraya	Mongrel (Dalmatiner)	Female	2	1	toy
Trulla	Border Collie	Female	1.5	1	toy
Bessy	Doberman	Female	2	1	toy
Sidney	Border Collie	Male	3	1	toy
Fara	Mongrel (Border Collie x German Shepherd)	Female	2	1	toy
Ronja	Mongrel (Labrador)	Female	2	1	toy
Piefke	Cocker Spaniel	Male	1.5	1	toy
Guenni	Whippet	Male	1	2	food
Maxl	Altdeutscher Fuchs	Male	2	2	food
Souris	Papillon	Female	2	2	food
Matilda	DSH, Mix	Female	1	2	food
Aimee	Collie	Female	2	2	food
Karou	Berger des Pyrenees	Male	4	2	food
Nilsson	Mix	Male	2	2	food
Tschaika	Mix, Sheltie, Spitz	Female	6	2	food
Laila	Golden Retriever	Female	2	2	food
Colin	Collie, Mix	Male	1	2	food
Sparky	Boxer, Mix	Male	1	2	food
Atze	Rauhaardackel	Male	5	2	food
Mali	Mix	Male	3	2	food
Felix	Mix	Male	5	2	food
Via	Dobermann	Female	1	2	food

Polly	Beagle	Female	1	2	food
Judy	Franz. Bulldogge	Female	2	2	food
Blue	Franz. Bulldogge	Male	2	2	food
Barry	Mix	Male	3	2	food
Kitana	Mix, Dobermann, DSH	Female	6	2	food
Feli	Jack Russel	Female	4	2	food
Mira	Podenco, Magyar Vizsla	Female	4	2	food
Migo	Jack Russel	Male	1	2	food
Daisy	Boerbel	Female	5	2	food
Bill	Golden Retriever	Male	7	3	food
Caesar	German Shepherd	Male	8	3	food
Cero	German Shepherd	Male	1	3	food
Cora	Bavarian Mountain Hound	Female	6	3	food
Corgo	Malinois	Male	2	3	food
Elsie	Mongrel	Female	5	3	food
Emma	Dalmatian	Female	3	3	food
Emma	Golden Retriever	Female	2	3	food
Hugsy	Husky	Female	1	3	food
Juna	Mongrel	Female	1	3	food
Kajsa	Australian Shepherd	Female	7	3	food
Krümel	Mongrel	Female	10	3	food
Lobo	Mongrel	Male	3	3	food
Matilda	Mongrel	Female	1	3	food
Mojo	Staffordshire Bullterrier	Female	2	3	food
Nana	Mongrel	Female	4	3	food

Nikita	Border Terrier	Female	1	3	food
Pantali	Malinois	Female	6	3	food
Paula	Podenco	Female	8	3	food
Poldi	Mongrel	Male	4	3	food
Sidi	Mongrel	Male	12	3	food
Skottie	Spaniel	Male	7	3	food
Spike	Mongrel	Male	11	3	food
Willy	Golden Retriever	Male	6	3	food
Yoshi	Mongrel	Male	10	3	food

Table 2. Overview of total number of sessions, sessions per testing day, trials per session and total number of trials listed for the three experiments.

	**Exp. 1**	**Exp. 2**	**Exp. 3**
Number of sessions	4	4	6
Sessions per testing day	2	2	3
Trials per session	12	16	24
Total number of trials	48	64	48

Table 3. Time variations for the two conditions (U = Unblocked trials, B = Blocked trials) and four different time delays (5, 20, 60, and 120 s) resulting in eight possible types of trials in Experiment 3.

**Delay**	**Unblocked**	**Blocked**
5	5U	5B
20	20U	20B
60	60U	60B
120	120U	120B

Table 4. Overview of the Coding Scheme including Checking, Correct/Incorrect choices, and Latency

Term	Definition
**Checking**	It was coded whether dogs checked through the outside of the fence structure. More precisely, we looked for any behavior of looking, sniffing, or scratching, which had to occur less than 10 cm from the gap in combination with hesitating, i.e. pausing at least half a second before selecting a side. Furthermore, we looked at which side the subjects checked before choosing one side and whether the chosen side was correct or not.
**Correct choice**	A correct choice, which also had to be the dog's first choice, was defined as the chosen barrier where the reward was hidden. The subject had to be at least with both front paws and the shoulder behind the outer side of the barrier where the food was baited.
**Latency**	We measured the latency between the starting signal including the release of the dog until the first choice, no matter if the choice was correct or incorrect. Furthermore, latency was only noted when there was no checking behavior in order to eliminate the additional time spent checking.

Supplemental Figures

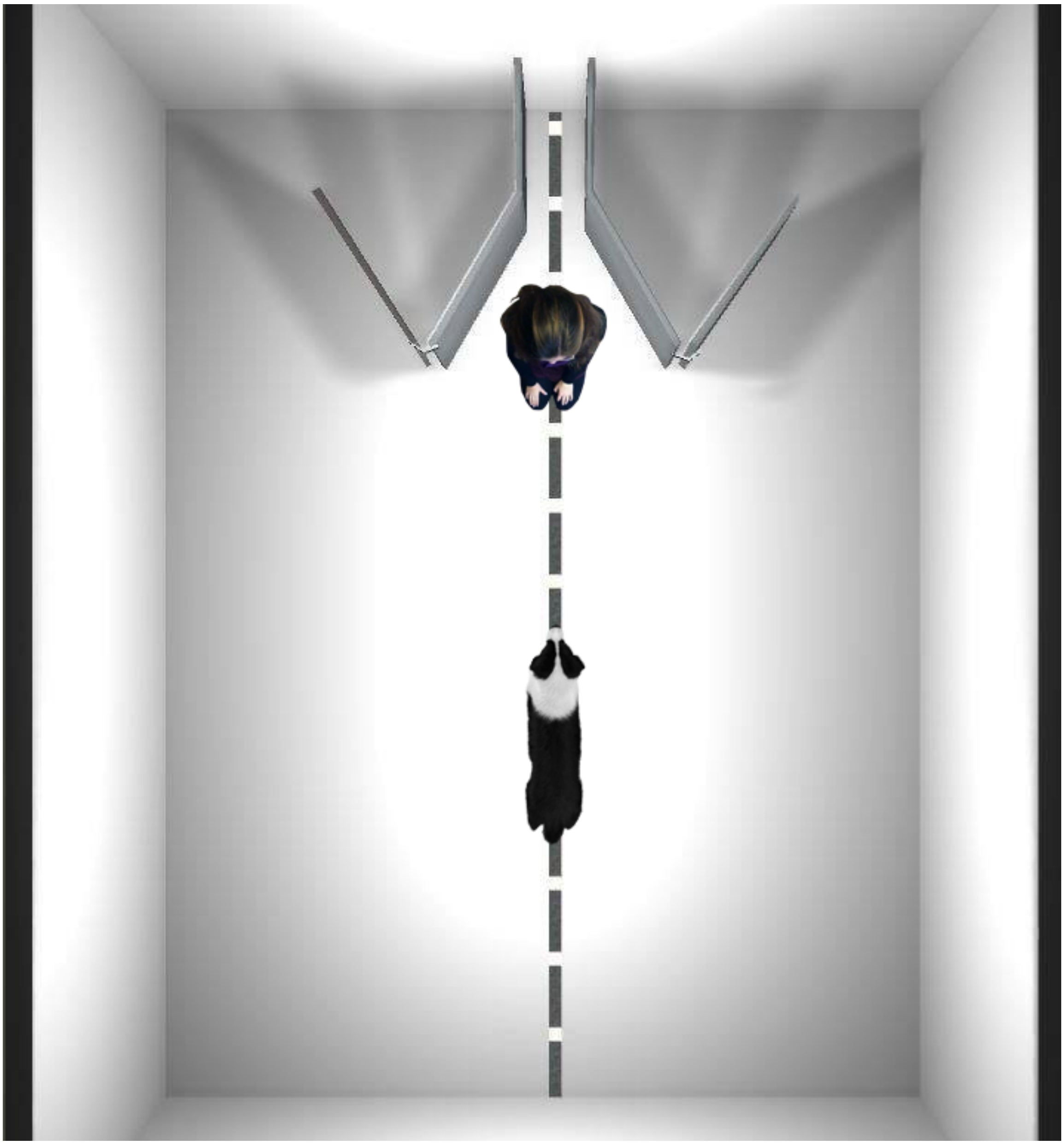

Figure 1. Experimental set-up depicted from the aerial perspective. The two-part apparatus consisted of two V-shaped wooden barriers (1.20 x 1.00 m) that were connected with a flexible hinge. The gap of approximately 2 cm allowed for checking.

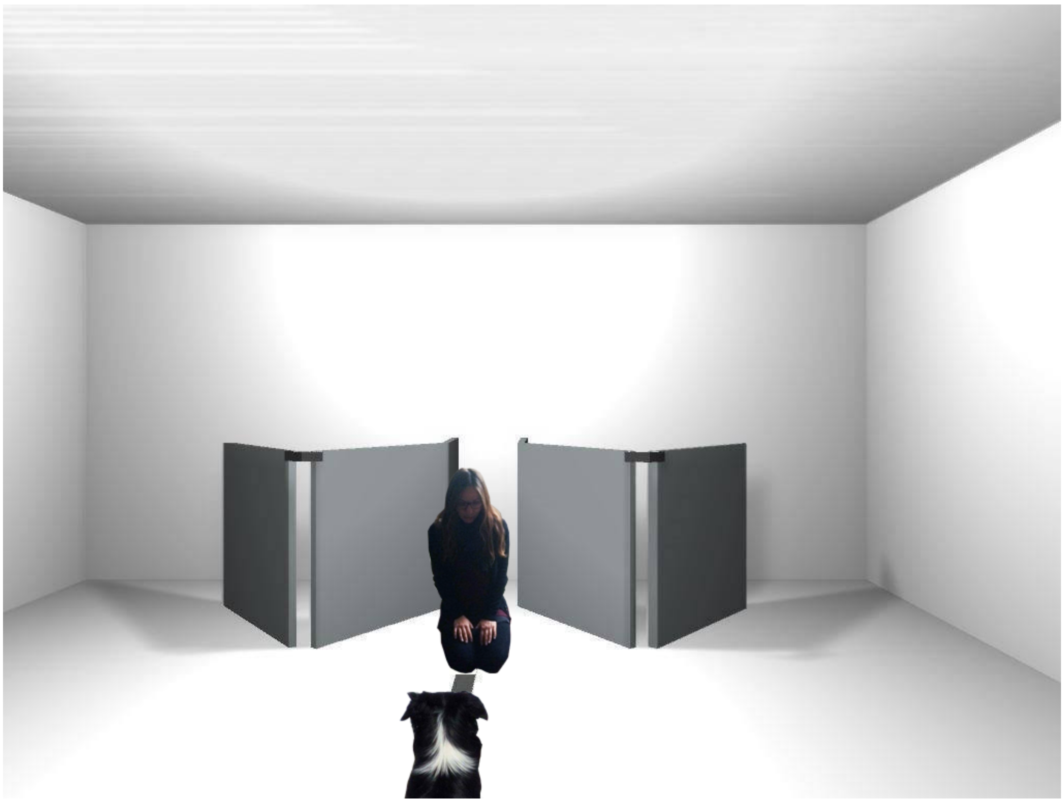

Figure 2. The experimental set-up of the apparatus from E2's perspective. As depicted, E1 sits in a neutral pose in the middle between both fences and does not give any nonverbal cues that could reveal the reward's location.

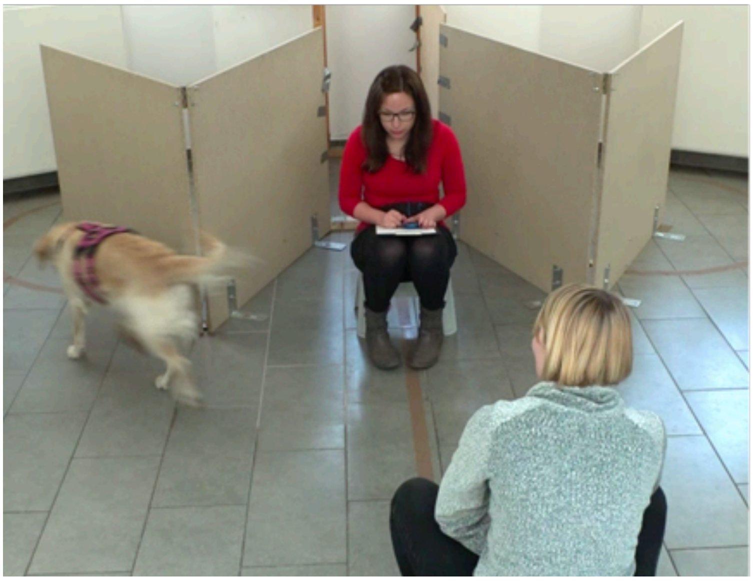

Figure 3. View from the camera of a dog selecting one side.
